# Supplementary material for: Double ridge formation over shallow water sills on Jupiter’s moon Europa
Source: Nat Commun. 2022 Apr 19;13:2007. doi: 10.1038/s41467-022-29458-3 (PMC9018861; doi:10.1038/s41467-022-29458-3)
Supplement: Supplementary file 1 — Supplementary Information [file 41467_2022_29458_MOESM1_ESM.pdf]

Supplementary Information for

Double Ridge Formation Over Shallow Water Sills on Jupiter's Moon Europa

Riley Culberg[1*], Dustin M. Schroeder[1,2], Gregor Steinbrügge[2]

[1] Department of Electrical Engineering, Stanford University, Stanford, CA, USA
[2] Department of Geophysics, Stanford University, Stanford, CA, USA

[*] Corresponding author: culberg@stanford.edu

This PDF file contains:

Supplementary Figures 1-6
Supplementary Table 1
Supplementary Methods

Supplementary Figures

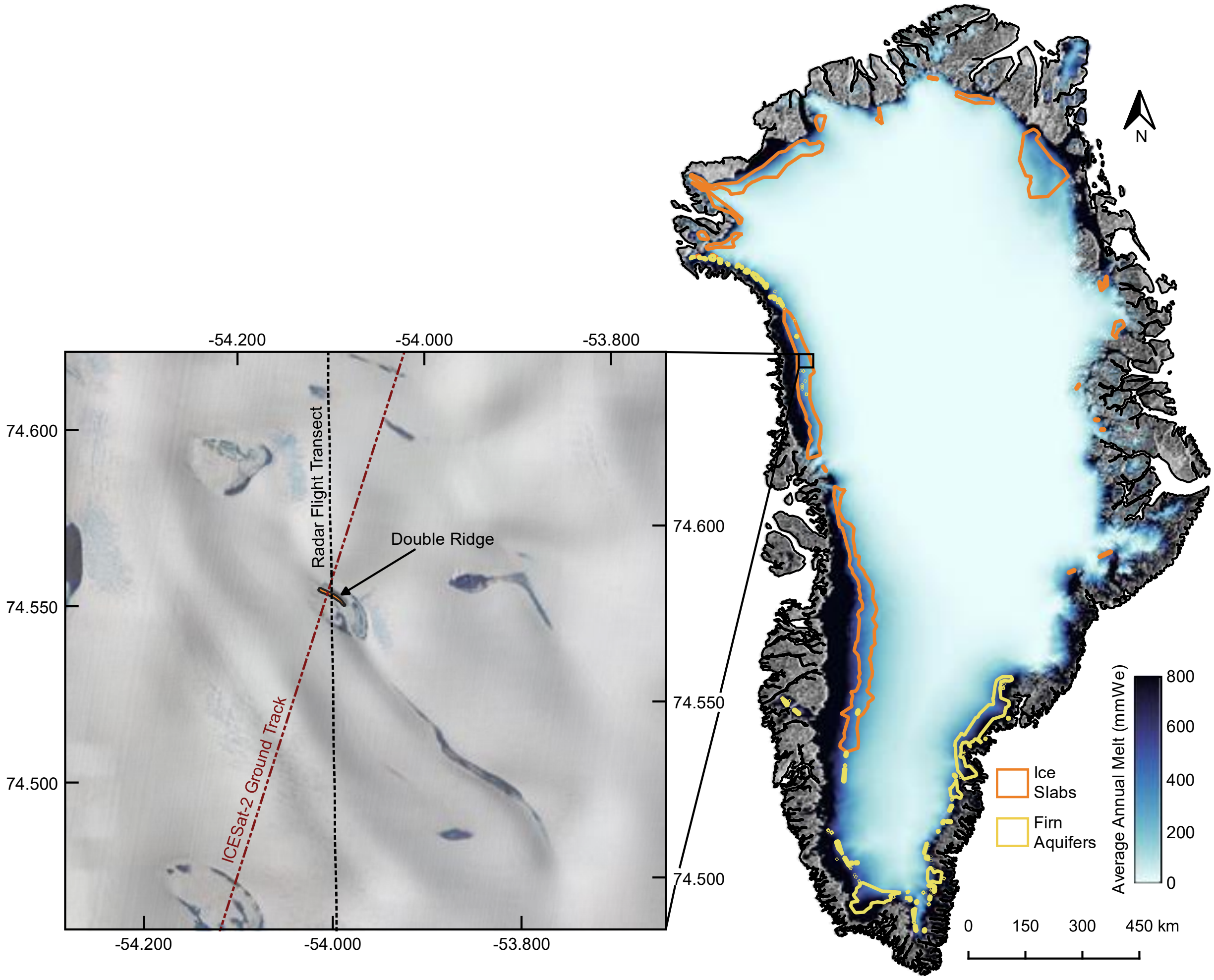

Supplementary Figure 1. Greenland context map. The Greenland map color scale represents average annual meltwater production as modeled by the regional climate model RACMO[1], with darker colors indicating more melt. Areas covered by ice slabs[2] and firn aquifers[3] are outlined in orange and yellow respectively. The inset map on the left shows surface topography from the ArcticDEM[4] 32 m composite hillshade with Sentinel-2 visible spectrum imagery from August 17, 2019 overlaid. The orange polygon outlines the location of the double ridge, centered at 74.566° N, 54.0531° W. The flight transect for all radar data (Fig. 2 and Fig. 3a-c) is marked in the dashed black line. The ICESat-2 ground track is shown in the dashed red line (Fig. 3d).

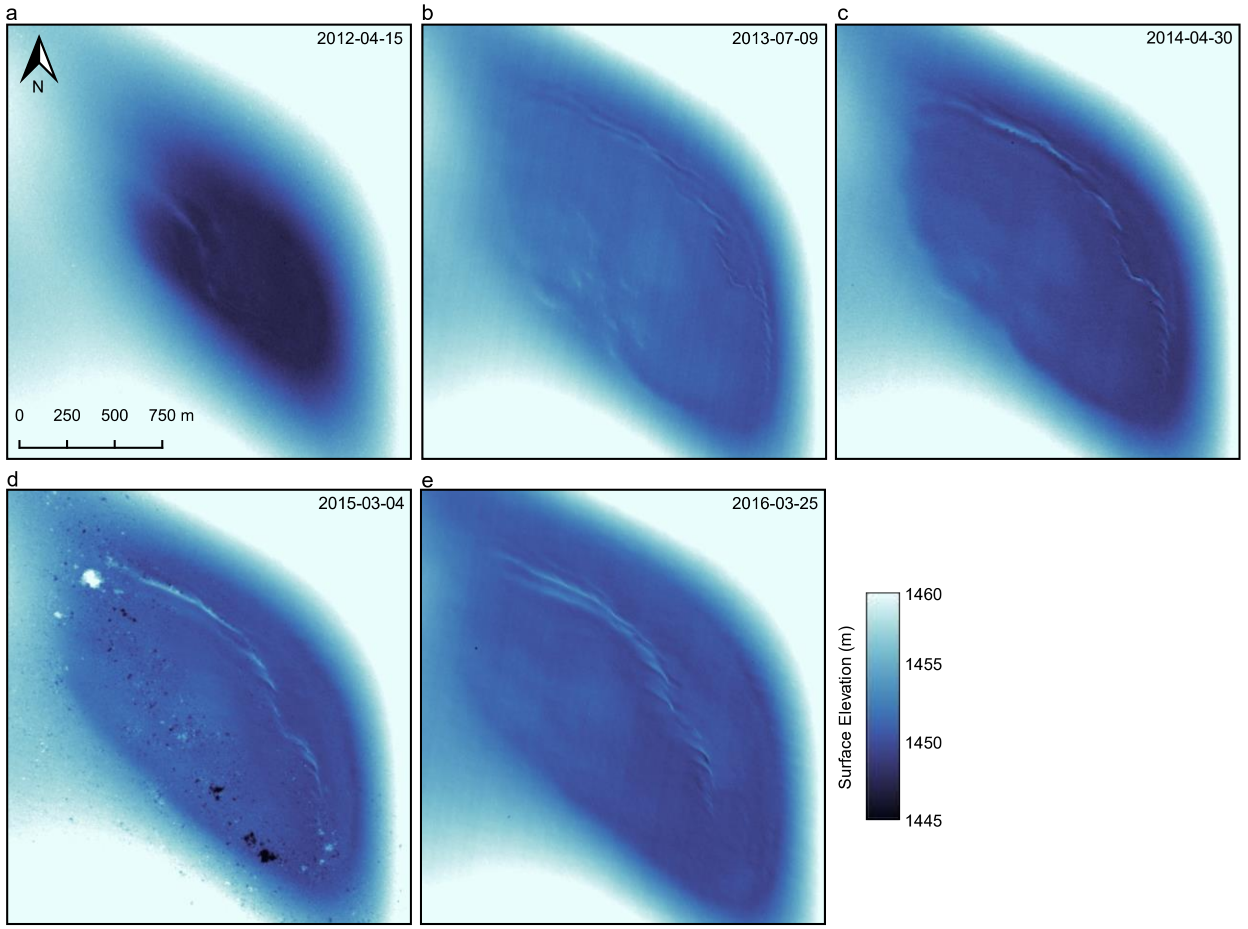

Supplementary Figure 2. Time series of ArcticDEM[4] surface elevation measurements over the double ridge. The color scale shows absolute surface elevation with darker colors indicating lower elevations. (a) April 2012. The ridge has not yet formed within the topographic basin. (b) July 2013. Significant surface uplift has occurred within the basin and an initial fracture is visible along the northern edge of the uplifted region. (c) April 2014. An asymmetric ridge system has developed around the initial fracture. (d) March 2015. The asymmetric ridge system persists. (e) March 2016. This is the first observation of the full double ridge.

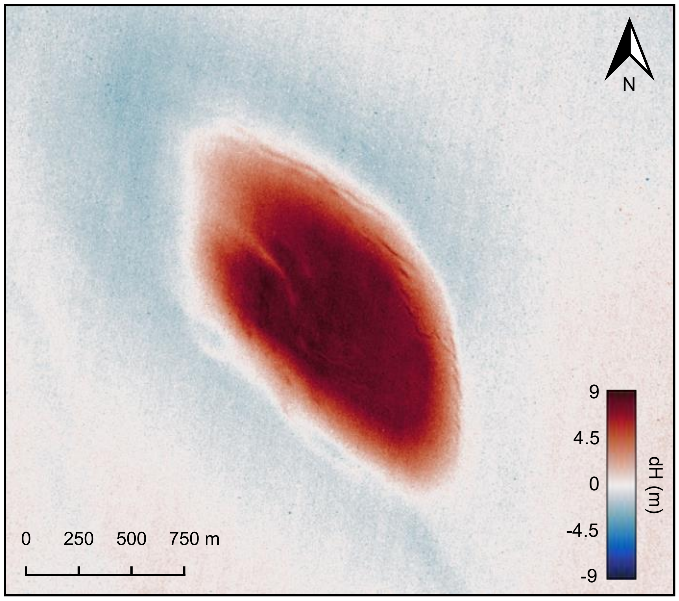

Supplementary Figure 3. ArcticDEM[4] surface elevation change between 15 April 2012 and 9 July 2013. Positive values shown in red indicate surface uplift and negative values shown in blue indicate surface subsidence. The uncertainty in elevation change is ±1.18 m.

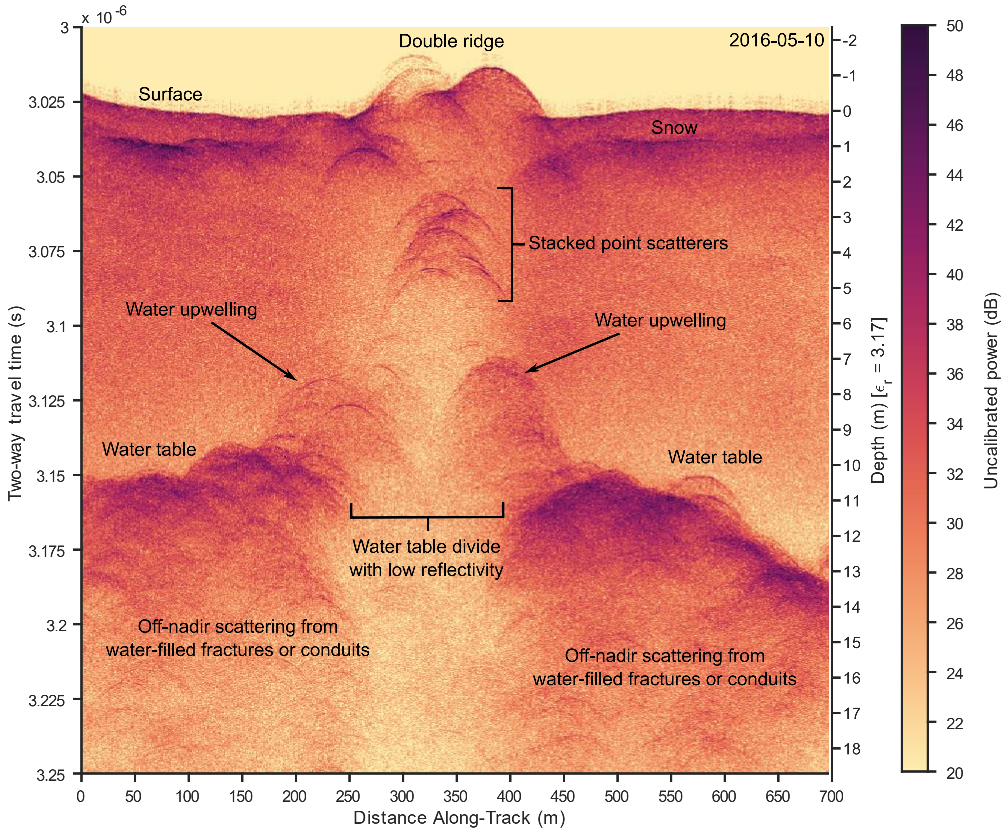

Supplementary Figure 4. Unfocused CReSIS Snow Radar data showing the double ridge and underlying divided water sill. Darker colors indicated strong radar returns. Hyperbolic returns, such as those from the stacked point scatterers beneath the central trough, typically indicate off-nadir scattering from rough surfaces or irregular volume geometries. We interpret the heavy off-nadir scattering at and beneath the water table as showing that the water is stored in a macro-porous fracture network. We interpret the stacked point scatterers beneath the central trough as scattering from irregular void space capping a central conduit or fracture network.

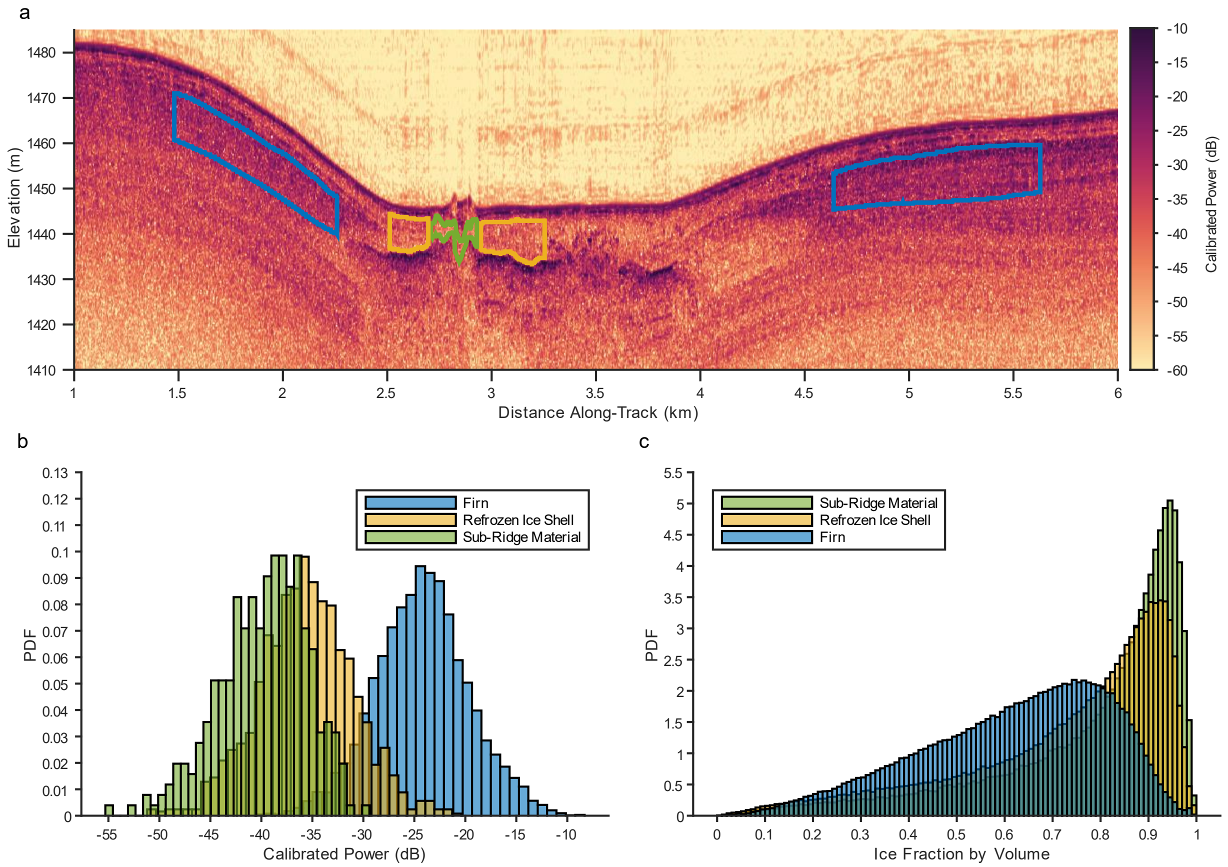

Supplementary Figure 5. Results of the MCMC inversion of radar reflectivity. (a) Radargram showing representative regions selected for inversion. Darker colors in the radargram indicate stronger radar returns. Regions outlined in blue are representative of firn. Regions outlined in yellow are representative of the homogeneous material encasing the sill. The region outlined in green represents the material beneath the ridge system. (b) Distributions of calibrated radar reflectivity within each region. (c) Distributions of volumetric ice fraction in each region as estimated by MCMC inversion of the reflectivity distributions presented in panel b.

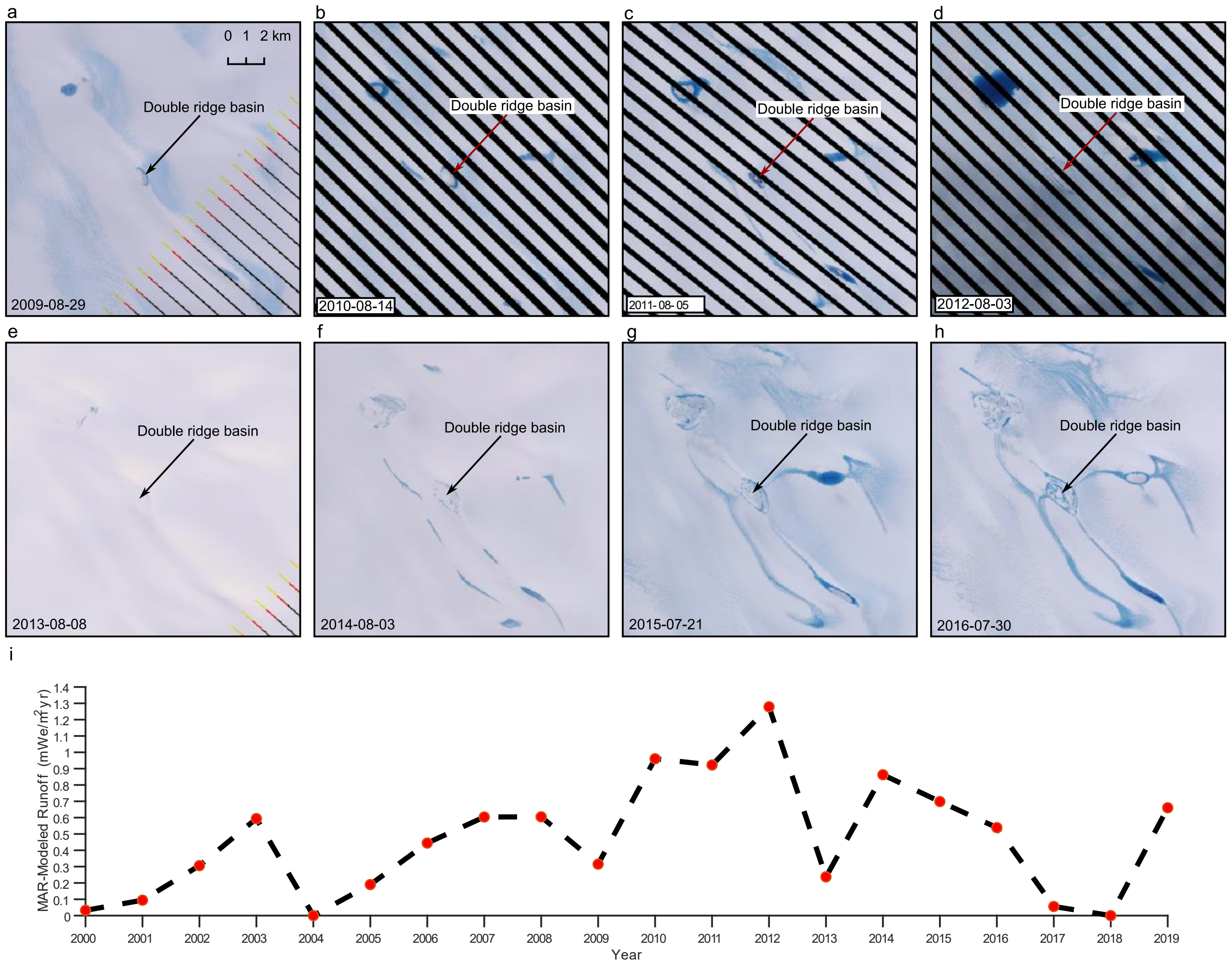

Supplementary Figure 6. Surface hydrology during double ridge formation. Panels a-e show Landsat 7 ETM+ true color optical satellite imagery of the basin containing the double ridge and surrounding areas at 30m spatial resolution. Black hashing in panels a-d is the result of the failure of the imaging system's scan line corrector. Panels f-h show Landsat 8 OLI true color optical satellite imagery of the same region at 30 m spatial resolution. This time series shows that surface streams, lakes, and slush fields are prominent at this location. Panel i shows the integrated annual surface runoff at this location as modeled by MARv3.10 from 2000 through 2019. Model values are shown in red dots, with the dashed black line showing a linear interpolation between model values. The lack of standing water in the double ridge basin in 2012 despite the record high modeled runoff suggests that runoff was routed to the subsurface, forming the sill later observed in the radar data.

Supplementary Tables

Supplementary Table 1. MCMC inversion and model parameters

**Model Parameters**	**Bounds**	**Step Size**
Mean Firn Density	0.45-0.75 gcm^{-3}	0.01 gcm^{-3}
Firn Density Variability	0.01-0.04 gcm^{-3}	0.001 gcm^{-3}
Melt Feature Percentage	0-1	0.03
Fractional Firn Area	0-1	0.03
**Observed Data Parameters**	**Mean Reflectivity**	**Uncertainty (σ)**
Firn	-24.35 dB	4.56 dB
Refrozen Ice Shell	-35.45 dB	4.31 dB
Sub-Ridge Material	-39.79 dB	4.26 dB
**Inversion Parameters**	**Value**	---
Number of Iterations	1,000,000	---
Proposal Distribution	Multivariate gaussian	---
Likelihood Function	Gaussian	---
Model Acceptance Ratio	53.6%-60%	---

Supplementary Methods

1. Datasets Used in This Study

ArcticDEM Surface Elevation Models

- SETSM_WV01_20120415_102001001A541000_1020010019508000_seg1_2m_v3.0
- SETSM_WV02_20130709_1030010024290300_1030010025350200_seg1_2m_v3.0
- SETSM_WV02_20140430_1030010030A71A00_103001002ECAD500_seg1_2m_v3.0
- SETSM_WV03_20150304_1040010008786500_1040010008387F00_seg2_2m_v3.0
- SETSM_WV03_20160325_104001001AA0CE00_104001001AB95600_seg1_2m_v3.0

ICESat-2 (ATL03 Global Geolocated Photon Data) Laser Altimeter Data

- photon_2018-10-20_t331_1603821655358 (gt11 beam)
- photon_2019-04-19_t331_1603821601489 (gt11 beam)
- photon_2020-01-17_t331_1603821383276 (gt11 beam)

CReSIS MCoRDS Radar Data

- 2015_Greenland_C130/20150512_02/Data_20150512_02_001-043
- 2016_Greenland_P3/20160510_01/Data_20160510_01_037
- 2017_Greenland_P3/20170327_02/Data_20170327_02_025

CReSIS Accumulation Radar Data

- 2017_Greenland_P3/20170327_04/Data_20170327_04_134-150
- 2017_Greenland_P3/20170328_01/Data_20170318_01_042-059

CReSIS Snow Radar Data

- 2015_Greenland_C130/20150501_02/Data_20150501_02_386
- 2016_Greenland_P3/20160510_15/Data_20160510_15_063-064
- 2017_Greenland_P3/20170327_03/Data_20170327_03_243

Commercial Satellite Imagery

- DigitalGlobe Catalog ID #1040010041897500

Galileo Images

- PIA00589

Landsat Imagery

- LE07_L1TP_018007_20090829
- LE07_L1TP_020007_20100814
- LE07_L1GT_016007_20110805
- LE07_L1TP_020007_20120803
- LE07_L1TP_018007_20130808
- LC08_L1TP_018007_20140803
- LC08_L1TP_018007_20150721
- LC08_L1TP_019007_20160730

2. Subsurface Scattering Properties

Absolute calibration using the scattered returns and radar forward model design require an understanding of the scattering properties of the subsurface material. In dry firn, reflections occur primarily due to the strong dielectric contrast at the interface between layers of different densities from different accumulation events[5,6]. At radar sounding frequencies and in nadir viewing geometries, volume scattering plays little to no role[7]. In regions of the Greenland Ice Sheet with high surface meltwater production, the subsurface density structure is modified by the infiltration and refreezing of meltwater, which may take the form of ice lenses or layers[8], ice pipes[9], and ice slabs[2,10]. These refrozen ice features have a strong density, and therefore permittivity, contrast with surrounding unmodified firn, resulting in high radar reflectivity. With the exception of vertically connecting ice pipes, the majority of refreeze features form in horizontal sheets due to the strong anisotropy of firn properties as stratigraphic layers are laid down by successive accumulation events. As a result, the subsurface geometry and structure, even in regions of high melt, can be modeled as stacked, specular reflecting facets within the radar footprint, rather than as a collection of point scatterers forming a volume scattering medium. However, in the case of extreme melt infiltration, this assumption is worth revisiting, particularly given the lack of clear stratigraphic interfaces in the radar data of interest.

A volumetric scattering medium produces diffuse scattering that is incoherent from one trace to the next whereas a stratified medium with sufficiently smooth interfaces produces scattering that exhibits some level of coherence from one trace to the next. Therefore, using the unfocused 2016 MCoRDS radargram, we calculate a horizontal coherence metric[11], modified from Oswald and Gogineni (2008), and given in Supplementary Equation 1.

$$I = \frac{\left|\int_X \Phi(x)dx\right|}{\int_X |\Phi(x)|dx} \qquad (1)$$

Here X is the horizontal length scale of interest and $\Phi(x)$ are the complex radar voltage measurements. In the case of perfectly coherent summation, the coherent (numerator) and incoherent (denominator) horizontal sums of radar traces will be equal and this ratio will be 1. In the case of perfect destructive interference between traces, this ratio becomes 0. Therefore, in a medium composed of specular facets whose length exceeds the along-track sampling distance of the radar system, we would expect a high coherence index. In a medium with a response dominated by random volume scattering at scales below the radar footprint, we would expect a low coherence index. By calculating this coherence index over various horizontal length scales, we can constrain the sub-resolution horizontal structure of the subsurface.

To compensate for phase variations between radar traces due to surface slope and aircraft motion, we flatten the radargram and apply a phase correction to each trace according to Supplementary Equation 2.

$$C_\phi = \exp\left(j\frac{4\pi}{\lambda}\Delta h\right) \qquad (2)$$

Here λ is the free-space wavelength of the radar and Δh is the difference in aircraft clearance over the surface between the first trace of the radargram and the trace being corrected. We then calculate the coherence index over length scales of 5 m, 10 m, 20 m, and 30 m and compare the results for radargram regions identified qualitatively as ice, firn, or noise. Ice regions include the ice slab and refrozen ice shell identified in Fig. 2, the firn consists of the region below the ice slab but shallower than 30 m depth, excluding the water table and refrozen ice shell (see Fig. 2), and the noise region consists of all data in the radargram at depths between 70 m and 125 m where no structure is

identifiable in the data. This noise region likely also samples some amount of surface and subsurface clutter, but we expect clutter to be a largely incoherent phenomenon as well.

At a length scale of 5 m, the median coherence (interquartile range given in parentheses) is 0.75 (0.37) for ice, 0.53 (0.39) for firn, and 0.2 (0.16) for noise. At 30 m, the scale of the first Fresnel zone, the median coherence is reduced to 0.42 (0.35) for ice, 0.23 (0.22) for firn, and 0.09 (0.07) for noise. The relatively high coherence at short length scales and reduced coherence at the Fresnel zone scale supports our interpretation that the sub-resolution horizontal structure consists of specular facets on the scale of a few meters in length and does not exhibit a volume scattering response despite the lack of clear stratigraphic structure in the power-detected radargrams.

3. Absolute Calibration of Radar Data

We absolutely calibrate the received radar power so that it can be compared directly to our analytical electromagnetic model. This involves correcting the received power, P_R, for geometric spreading (G), attenuation (A), rough interface scattering loss (L), birefringence loss (B), and some constant radar system offset (S) [12]. Supplementary Equation 3 describes the relationship between these variables and the received and calibrated power (P_C) in decibels.

$$\lfloor P_c \rfloor_{dB} = \lfloor P_R \rfloor_{dB} + \lfloor G \rfloor_{dB} + \lfloor A \rfloor_{dB} + \lfloor L \rfloor_{dB} + \lfloor B \rfloor_{dB} + \lfloor S \rfloor_{dB} \quad (3)$$

We geometrically correct the received power according to Supplementary Equation 4.

$$G = 20 \log_{10} \left[2 \left(h + \frac{d}{1.78} \right) \right] \quad (4)$$

Here h is the aircraft height above the surface, d is the depth below the surface, and 1.78 is the refractive index of solid ice. Varying the estimated index of refraction between 1.5 and 1.78 (bulk densities of 0.6 gcm^{-3} and 0.917 gcm^{-3}) results in variations in the geometric correction value of less than 0.1 dB for a typical flight altitude of 500 m and layer depth of 30 m, which is negligible compared with other sources of uncertainty. Based on our assumption that the reflectivity is dominated by specular facets within the footprint, we assume a range-squared dependence for this geometric correction[13].

We estimate the englacial attenuation based on firn conductivity measurements and temperature profiles[14]. We use firn conductivity measurements from the B26-B29 firn cores from the North Greenland Traverse[15–18], which are given relative to a common temperature of -15 °C. We correct the conductivity as a function of depth for the true ice sheet temperature profile using the Arhenius relationship[14] in Supplementary Equation 5.

$$\sigma_c[z] = \sigma_m[z] \exp \left[\frac{E}{R} \left(\frac{1}{T_m} - \frac{1}{T[z]} \right) \right] \quad (5)$$

Here σ_m is the measured conductivity as a function of depth z, E is the activation energy, R is the gas constant, T_m is the calibrated temperature at which the measurements were published, $T[z]$ is the modeled true temperature as a function of depth, and σ_c is the corrected conductivity. We recalibrate the pure ice and impurity components of conductivity separately with activation energies of 0.5 eV and 0.22 eV respectively[19]. We use the modeled subsurface temperature profile in May 2016 from MARv3.5.2[20]. However, there is typically a large degree of uncertainty in modeled firn temperature profiles, particularly if the effects of latent heat from subsurface water storage are not well accounted for. Therefore, we consider two additional bounding cases, one where the subsurface temperature is uniformly at the mean annual surface temperature of -14 °C and one where the temperature is uniformly at 0 °C. The first places a lower bound on the attenuation, and the second, an upper bound. We calculate the total attenuation in dB as a function depth according to Supplementary Equation 6.

$$A[z] = 8.686 \sum_{i=0}^{N} \Delta z_i \frac{\sigma_i}{2} \sqrt{\frac{\mu_o}{\epsilon_o \epsilon_i}} \quad (6)$$

Here Δz_i is the thickness of each subsurface layer, σ_i is the corrected conductivity of that layer, μ_o is the magnetic permeability of free space, ϵ_o is the electric permittivity of free space, and ϵ_i is the relative permittivity of the layer, which we assume to be 3.17. We separately correct the received radar power using each attenuation model and compare the distributions of radar reflectivity in each region delineated in Supplementary Fig. 5. We find that there is no statistically significant shift in the distributions as a result of using a different attenuation model. In all cases, the new distribution means calculated after correction with a -14 °C or 0 °C temperature profile fall within the bootstrapped confidence intervals on the means of the distributions calculated using the MARv3.5.2 correction. This is due to the shallow depths of the features of interest – total attenuation ranges from 0 dB at the surface to 1.6 dB at a depth of 30 meters in the warmest scenario, with a maximum difference of only ~1 dB between the warmest and coldest models. Therefore, we correct for attenuation using only the MARv.3.5.2 temperature profile in all further analysis.

Accounting for scattering loss is challenging as it requires a strong knowledge of the subsurface geometry and its scattering properties. In order to minimize these losses, we SAR focus the radargrams in order to migrate power back to scattering centers. However, this remains a source of uncertainty in our calibration. We also neglect birefringence loss as it is typically assumed to be small in the near-surface[21].

The radar system correction is applied as a single offset to each transect. For absolute calibration, we solve for the system offset that minimizes the mean square error between our radar observations and a model of theoretical reflectivity calculated from high resolution firn density measurements input to a 1D layered dielectric electromagnetic model[8]. In this case, the flight transect of interest does not directly intersect one of the sites with adequate firn density measurements. Therefore, we first cross-level[22] the flight line of interest with other flight lines that can be directly calibrated at one of these sites. Unfortunately, no flight lines from the 2016 MCoRDS season meet this criterion. Therefore, we calibrate the flight line twice. In the first, we use a single crossover with data collected in 2015 at a similar center frequency of 315 MHz. In the second, we cross-level with data collected by the Accumulation Radar in 2017 which operates at a much higher frequency of 750 MHz, but was a direct repeat of our flight line of interest. We choose the Accumulation Radar data rather than the coincident MCoRDS data because the 2017 Accumulation Radar data has approximately the same range resolution as the 2016 MCoRDS data, thereby minimizing the impact of integrating a greater subsurface region into the surface return. By observing the difference in the estimated system constants from each calibration, we can estimate the uncertainty in our calibration due to the non-ideal properties of the tie-lines.

To cross-level these transects, we extract the surface power, excluding data points where aircraft roll exceeds 0.05 radians, as this corresponds to the point at which mean surface power falls approximately 1 dB below average due to antenna beam pattern suppression. We then solve for an initial offset that minimizes the mean square error in surface power at all crossovers points. We absolutely calibrate the 2015 tie-line with the B16 firn core[23] and 2017 tie-line with the B29 firn core[24]. We find that calibration with the 2015 tie-line gives $S = -16.3$ dB and, with the 2017 tie-line, $S = -16.8$ dB. Given that there is only a 0.5 dB difference in the system constant despite calibration across different seasons with different firn cores, we assess that our calibration is stable and minimally effected by the non-ideal tie-lines. The average calibrated surface reflectivity is

consistent with a surface density of 0.35 gcm^{-3}, the average surface density of the Greenland Ice Sheet[25], suggesting that our calibrated reflectivities also fall within a physically reasonable range. We ultimately use an average system constant of -16.55 dB to correct the radargrams.

A potential source of calibration error is the lack of interface roughness effects in our 1D model, which could result in an overestimation of the system offset. Our calibration matches the modeled specular subsurface reflectivities at the B29 and B16 firn core sites, where we have previously estimated the firn layer roughness to be approximately 3 cm[7]. The system constant therefore includes an implicit correction for ~3 cm of interface roughness. Since our calibration cores are located in regions of the ice sheet with the smoothest surface[25] and ice layers may be roughened by ice glands, pipes, and heterogeneous refreezing[26], we expect that this correction is an underestimation of interface roughness in our regions of interest and therefore still a conservative calibration approach. This means that the reported reflectivity values are may be somewhat lower than the true material reflectivity. When inverted, this would result in a dampened estimate of the permittivity contrast.

4. Radar Forward Model

Based on the physical observations and radar scattering character, we employ a radar forward model that assumes the subsurface can be described as a collection of reflecting firn facets embedded in refrozen ice. The objective of this model is to simulate the effective reflectivity of the illuminated subsurface volume that would contribute to single measured peak in the observed data. Therefore, the volume of interest is bounded by the range and azimuth resolutions of the radar system.

We assume that the volume of this radar footprint consists of refrozen ice with a uniform reflectivity of -67 dB or roughly equivalent to a 0.005 gcm^{-3} variability in density within the homogeneous ice regions. This value represents an approximate crossover point below which variations in ice conductivity would have a greater contribution to the reflectivity than the variation in density. Therefore, it approximates the highest reflectivity we might expect for solid ice. Some fractional area of the footprint is also occupied by relict firn patches. These patches are assumed to have a diameter greater than or equal to twice the wavelength of the radar (~1 meter in ice), as our model treats each patch as a reflecting facet, rather than a scattering point source, and neglects edge effects. The total patch height is set equal to the radar range resolution (~0.42 m in ice for this system). The vertical structure of each patch can be described by a 1D vertical density profile that represents the stratigraphic structure of the firn. Some of these layers within the firn patch may also consist of refrozen ice. Clearly, this is a complicated structure which we cannot expect to deterministically define, given that there may be tens of layers in each firn patch. Therefore, we use a stochastic model to simulate density profiles for these firn patches[27]. This stochastic model is parameterized by mean firn density, firn density variability, and melt feature percentage (MFP) – the total fractional height of refrozen ice layers within the firn patch. We use an autoregressive random process trained on high resolution firn cores from the North Greenland Traverse to simulate the dry firn density profile[27]. We then recursively add ice layers to this profile, drawing their thickness and spacing from uniform distributions over the remaining height of the patch, until the MFP parameter is satisfied. Therefore, the structure of the radar footprint can be described as a collection of firn patches occupying some total fractional area, A_f, each with a randomly generated density profile parameterized on mean firn density, firn variability, and MFP. We calculate the effective reflectivity of each firn patch by converting the density profile to refractive index using the Kovacs relationship[28] and apply our 1D layered dielectric EM model[27] to calculate

the reflectivity of each patch, Γ_i, using a transfer matrix-based approach. Following the Berry (1973)[29] and Peters, et al (2005)[30] formulation for the net reflection coefficient of a radar resolution cell with spatially varying material properties, the effective reflectivity of the entire radar footprint is then given by Supplementary Equation 7.

$$|\Gamma_e|^2 = \left|\sum_{i=1}^{N}\left(\frac{A_i}{A_{tot}}\right)\Gamma_i\right|^2 \tag{7}$$

Here, Γ_e is the effective reflectivity of the footprint, N is the number of firn and ice patches, A_i is the surface area of each patch, A_{tot} is the total surface area of the radar footprint, and Γ_i is the reflectivity of each patch.

We invert radar data that was SAR focused with a target azimuth resolution of 2.5 m and assume that the total scattering area is a rectangle with a width set by the azimuth resolution and a length set by the diameter of the first Fresnel zone. Supplementary Table 1 lists the bounds we place on the model parameters. The bounds on mean firn density are set based on the range of densities observed in firn cores from ice slab areas in Southwest Greenland[10,31]. The bounds on firn density variability are set based on measurements from North Greenland Traverse firn cores. We use this data from other regions of the ice sheet as there no publicly available firn core data for the Northwest Greenland percolation zone in the last 50 years, and the North Greenland Traverse cores represent the only cores on the ice sheet with sufficient measurement resolution to capture the centimeter-scale variability in density.

5. Radar Inversion Sensitivity Tests

We run a series of sensitivity tests to assess the robustness of our estimated distributions of fractional volumetric ice content. We use the same MCMC inversion parameters as the initial inversion, but only run 500,000 iterations for more rapid testing. In our original model, we assumed that the focused footprint can be approximated as a rectangle whose width is set by the azimuth resolution, length is set by the diameter of the first Fresnel zone, and height is set by the radar range resolution. In the first test, we instead use a circular scattering area equal to the first Fresnel zone. While perfect SAR migration should restrict the footprint width to the target azimuth resolution, the true effective scattering area is more uncertain in real data due to phase errors and limited scattering apertures for quasi-specular reflectors. Therefore, we test a bounding case in which the focused footprint consists of a cylinder with the radius set by the radius of the first Fresnel zone and the height equal to the radar range resolution. From this test, we estimate a firn ice fraction of 0.67 (0.27) (median and interquartile range), refrozen ice shell ice fraction of 0.8 (0.27), and median sub-ridge material ice fraction of 0.85 (0.23). These values are consistent with the initial estimates of 0.65 (0.28), 0.8 (0.27), and 0.86 (0.23) respectively from our baseline inversion.

In the second test, we consider the possible impact of variable surface roughness on the reflectivity calculations. Surface roughness can induce power loss in subsurface returns due to small phase variations induced at the interface that reduce the coherence of the subsurface wavefront[32]. As the region of apparent lowest reflectivity also falls directly beneath the ridges, which are the largest source of local surface roughness, this could be an important confounding factor in our interpretation of the subsurface reflectivity. We first estimate the local surface roughness from the 2016 ArcticDEM data[4]. At the center point of each radar trace, we extract elevation profiles spanning the first Fresnel zone parallel and perpendicular to the flight line, detrend each with a linear fit, and calculate the root mean square (RMS) height for each detrended

profile. We average the along-track and across-track RMS heights to produce a single estimate at each trace. We then calculate a mean RMS height for the surface profile spanning each of the regions selected as representative of firn, refrozen ice shell, and sub-ridge material in our radar data. In our inversion process, we then apply a random phase offset to each patch within the footprint whose values are drawn from a zero-mean Gaussian distribution whose standard deviation is set by this average RMS height. The RMS height to phase transform applied to the ith patch is given in Supplementary Equation 8.

$$\phi_i = \text{mod}\left[\left(\frac{4\pi\sigma_h}{\lambda}\right)(n_{ice} - 1)x_i, 2\pi\right] \quad (8)$$

Here σ_h is the RMS height of the surface, λ is the free space wavelength of the radar system, n_{ice} is the index of refraction of ice (1.78), and $x_i \sim \mathcal{N}(0,1)$. This effectively models the surface roughness as a random phase screen applied to the incident wave.

After applying these phase variations, we see a small reduction in estimated fractional ice content with a firn ice fraction of 0.64 (0.28), median refrozen ice shell ice fraction of 0.78 (0.27), and median sub-ridge material ice fraction of 0.83 (0.27). However, the relative difference between the distributions for each region are preserved, suggesting that our interpretation that the firn has seen the least water infiltration and refreezing and the sub-ridge material has seen the most refreezing is robust to uncertainty in the surface roughness losses. In this scattering model, reflecting firn patches are generally not coherent with one another, as their physical structure varies within the footprint. As a result, their phase relationship is already effectively random and the additional phase screen does not materially change the distribution of phases within a single footprint. Therefore, the distribution of modeled reflectivities, and by extension ice fraction, does not change significantly.

In the final test, we alter our radar forward model to assume that the background material consists of layered firn, with homogeneous ice patches embedded within it. We otherwise implement this test in the same manner as the initial inversion. In this case, the MCMC inversion converges to a mirrored solution, with an estimated median firn ice fraction of 0.29 (0.27), median refrozen ice shell ice fraction of 0.15 (0.27), and median sub-ridge ice fraction of 0.12 (0.27). This mirroring likely occurs because in the two-dimensional MFP – firn fractional area space, the objective function maximum forms a u-shape, where, for example, either high MFP and high fractional firn area or low MFP and high fractional firn area can produce similarly low reflection coefficients. The original model formulation treats the background material as firn with an MFP of 100% (i.e., solid ice) where this alternate model treats the background as firn with an MFP of 0%. Therefore, the original model tends to converge towards the high MFP side of the solution curve and the alternate model converges towards the low MFP side.

This solution shows that one physical configuration that could produce the observed low radar reflectivities is dry firn with minimal ice layers or embedded ice patches. We can clearly rule out this solution space as physically unrealistic. For this to be true, the firn must have experienced the most modification by meltwater infiltration and the material surrounding the liquid water sill and beneath the ridges must have experienced almost no modification by refreezing. Given the massive liquid water storage we observe, it is implausible that the subsurface material immediately surrounding that water body has undergone a level of refreezing on par with that of the shallow percolation zone where a fraction of the annual melt occurs. Additionally, we would expect to see clear stratigraphic subsurface boundaries, as we do in the dry snow zone, if the subsurface structure was well modeled by a 1D density structure with a few ice layers and isolated ice patches.

Supplementary References

1. Noël, B. *et al.* Evaluation of the updated regional climate model RACMO2.3: Summer snowfall impact on the Greenland Ice Sheet. *Cryosphere* **9**, 1831–1844 (2015).
2. MacFerrin, M. *et al.* Rapid expansion of Greenland's low-permeability ice slabs. *Nature* **573**, 403–407 (2019).
3. Miège, C. *et al.* Spatial extent and temporal variability of Greenland firn aquifers detected by ground and airborne radars. *J. Geophys. Res. Earth Surf.* **121**, 2381–2398 (2016).
4. Porter, C. *et al.* ArcticDEM,V1. *Harvard Dataverse*. doi:10.7910/DVN/OHHUKH (2018).
5. Fujita, S. *et al.* Nature of radio echo layering in the Antarctic Ice Sheet detected by a two-frequency experiment. *J. Geophys. Res.* **104**, 13013–13024 (1999).
6. Eisen, O., Wilhelms, F., Nixdorf, U. & Miller, H. Revealing the nature of radar reflections in ice: DEP-based FDTD forward modeling. *Geophys. Res. Lett.* **30**, 1–4 (2003).
7. Culberg, R. & Schroeder, D. M. Firn Clutter Constraints on the Design and Performance of Orbital Radar Ice Sounders. *IEEE Trans. Geosci. Remote Sens.* **58**, 1–18 (2020).
8. Culberg, R., Schroeder, D. M. & Chu, W. Extreme melt season ice layers reduce firn permeability across Greenland. *Nat. Commun.* **12**, 1–9 (2021).
9. Humphrey, N. F., Harper, J. T. & Pfeffer, W. T. Thermal tracking of meltwater retention in Greenland's accumulation area. *J. Geophys. Res. Earth Surf.* **117**, 1–11 (2012).
10. Machguth, H. *et al.* Greenland meltwater storage in firn limited by near-surface ice formation. *Nat. Clim. Chang.* **6**, 390–393 (2016).
11. Oswald, G. K. A. & Gogineni, S. P. Recovery of subglacial water extent from Greenland radar survey data. *J. Glaciol.* **54**, 94–106 (2008).
12. Matsuoka, K., MacGregor, J. A. & Pattyn, F. Predicting radar attenuation within the Antarctic ice sheet. *Earth Planet. Sci. Lett.* **359**–**360**, 173–183 (2012).
13. Haynes, M. S., Chapin, E. & Schroeder, D. M. Geometric Power Fall-off in Radar Sounding. *IEEE Geosci. Remote Sens. Lett.* **56**, 6571–6585 (2018).
14. Zirizzotti, A., Cafarella, L., Urbini, S. & Baskaradas, J. A. Electromagnetic ice absorption rate at Dome C, Antarctica. *J. Glaciol.* **60**, 849–854 (2014).
15. Wilhelms, F. Conductivity of ice core ngt37C95.2 (B26) from the North Greenland Traverse. *PANGEA*. doi: 10.1594/PANGAEA.58011 (2001).
16. Wilhelms, F. Conductivity of ice core ngt39C95.2 (B27) from the North Greenland Traverse. *PANGEA*. doi:10.1594/PANGAEA.58012 (2001).
17. Wilhelms, F. Conductivity of ice core ngt39C95.3 (B28) from the North Greenland Traverse. *PANGEA*. doi:10.1594/PANGAEA.58013 (2001).
18. Wilhelms, F. Conductivity of ice core ngt42C95.2 (B29) from the North Greenland Traverse. *PANGEA*. doi:10.1594/PANGAEA.58014 (2001).
19. Wolff, E. W. *et al.* Long-term changes in the acid and salt concentrations of the Greenland Ice Core Project ice core from electrical stratigraphy. *J. Geophys. Res.* **100**, 16,216-249,263 (1995).
20. Fettweis, X. *et al.* Reconstructions of the 1900 – 2015 Greenland ice sheet surface mass balance using the regional climate MAR model. *Cryosph.* **11**, 1015–1033 (2017).
21. Fujita, S., Maeno, H. & Matsuoka, K. Radio-wave depolarization and scattering within ice sheets: A matrix-based model to link radar and ice-core measurements and its application. *J. Glaciol.* **52**, 407–424 (2006).
22. Chu, W. *et al.* Extensive winter subglacial water storage beneath the Greenland Ice Sheet.

Geophys. Res. Lett. **43**, 12,484-12,492 (2016).
23. Wilhelms, F. Density of ice core ngt03C93.2 (B16) from the North Greenland Traverse. *PANGEA*. doi:10.1594/PANGAEA.56560 (2000).
24. Miller, H. & Schwager, M. Density of ice core ngt42C95.2 (B29) from the North Greenland Traverse. *PANGEA*. doi: 10.1594/PANGAEA.57655 (2000).
25. Van Der Veen, C. J., Krabill, W. B., Csatho, B. M. & Bolzan, J. F. Surface roughness on the Greenland ice sheet from airborne laser altimetry, **25** (12), 3887–3890 (1998).
26. Brandt, O., Langley, K., Giannopoulos, A., Hamran, S. E. & Kohler, J. Radar response of firn exposed to seasonal percolation, validation using cores and FDTD modeling. *IEEE Trans. Geosci. Remote Sens.* **47**, 2773–2786 (2009).
27. Culberg, R. & Schroeder, D. M. Strong Potential for the Detection of Refrozen Ice Layers in Greenland's Firn By Airborne Radar Sounding. in *IGARSS 2020*, 7033–7036 (2020).
28. Kovacs, A., Gow, A. J. & Morey, R. M. A reassessment of the in-situ dielectric constant of polar firn. *CRREL Report 93-26*, (1993).
29. Berry, M. V. The statistical properties of echoes diffracted from rough surfaces. *Philos. Trans. R. Soc. A Math. Phys. Eng. Sci.* **273**, 611–658 (1973).
30. Peters, M. E., Blankenship, D. D. & Morse, D. L. Analysis techniques for coherent airborne radar sounding: Application to West Antarctic ice streams. *J. Geophys. Res. Solid Earth* **110**, 1–17 (2005).
31. Koenig, L. S. & Montgomery, L. N. Surface Mass Balance and Snow Depth on Sea ice Working Group (SUMup) accumulation on land ice subdataset, Greenland and Antarctica, 1987-2018. (2018) doi:doi:10.18739/A2ZS2KD0Z.
32. Schroeder, D. M., Grima, C. & Blankenship, D. D. Evidence for variable grounding-zone and shear-margin basal conditions across Thwaites Glacier, West Antarctica. *Geophysics* **81**, WA35–WA43 (2016).
